# Mpox Epidemiology and Vaccine Effectiveness, England, 2023

**DOI:** 10.3201/eid3010.240292

**Published:** 2024-10

**Authors:** Hannah Charles, Katie Thorley, Charlie Turner, Kirsty F. Bennet, Nick Andrews, Marta Bertran, Sema Mandal, Gayatri Amirthalingam, Mary E. Ramsay, Hamish Mohammed, Katy Sinka

**Affiliations:** UK Health Security Agency, London, UK

**Keywords:** mpox, monkeypox virus, surveillance, epidemiology, men who have sex with men, GBMSM, vaccine effectiveness, viruses, England

## Abstract

Reported mpox cases in England continued at a low but steady frequency during 2023. Of 137 cases reported in 2023, approximately half were acquired overseas and half were in vaccinated persons. Estimated effectiveness of 2-dose vaccine was 80%, and no vaccinated mpox patient was hospitalized.

In England, after the July 2022 peak in the mpox outbreak (*1*), which affected primarily gay, bisexual, and other men who have sex with men (GBMSM), cases declined and remained low into 2023 (*2*). We analyzed the epidemiology of postpeak mpox cases in 2023 in England, describing case-patient characteristics including vaccination status and providing an updated estimate of Modified Vaccinia Ankara–Bavarian Nordic (MVA-BN) vaccine effectiveness (VE). 

## The Study

We extracted records of confirmed and highly probable mpox diagnoses reported during January 1–December 31, 2023, from the UK Health Security Agency (UKHSA) Second Generation Surveillance System (SGSS) and deduplicated them by using specimen and patient identifiers. We defined a confirmed case as a positive monkeypox virus–specific PCR result and a highly probable case as a positive *Orthopoxvirus* PCR result (*3*). SGSS receives positive test results from all diagnostic laboratories in England (*4*). Because mpox is notifiable in England (*5*), reporting to SGSS is probably complete. UKHSA local Health Protection Teams collected self-reported epidemiologic and behavioral information (including vaccination status). NHS England provided aggregate data for mpox vaccinations administered in 2022 and 2023. We estimated overall VE by using the screening method (*6*). For the primary VE analysis, the eligible GBMSM denominator was 89,240 and was 20% higher at 107,088 according to a sensitivity analysis, similar to previous analyses (*7*). We estimated the proportion of GBMSM vaccinated by matching each case to the 1- and 2-dose coverage at the time, 2 weeks before persons became case-patients, then averaging the matched coverage across cases. For the 22 case-patients for whom vaccination status was unknown, we assumed that they would be distributed among those with 0, 1, and 2 doses in the same ratio as the observed ratio for these groups, to give a corrected value. We tested the difference between 1-dose and 2-dose VE estimates by using the Pearson χ^2^ test at 5% significance.

During January 1–December 31, 2023, a total of 137 mpox cases were reported (Appendix Figure), 135 confirmed and 2 highly probable. Most case-patients (105/137 [77%]) were London residents, comparable to case-patients in 2022 (Table 1). Of the 137 case-patients, 64 (47%) reported no travel outside the United Kingdom in the 21 days before symptom onset, indicating probable acquisition in the United Kingdom; 58 (91%) of the 64 identified as GBMSM. Of the 137 case-patients, 53 (39%) reported international travel, among which 43 (81%) identified as GBMSM and reported traveling to Europe, the Middle East, Asia Pacific region, and North America.

**Table 1:** Available demographic characteristics of mpox case-patients, England, 2022 and 2023*

Variable	Year, no, (%)	
	2022, n = 3,412†	2023, n = 137
Region		
London	2,359 (69)	105 (77.0)
Outside London	1,040 (30)	32 (23.0)
Unknown	13 (0.4)	0
Sex		
M	3,345 (98)	135 (99.0)
F	45 (1.3)	1 (0.7)
Unknown	22 (0.6)	1 (0.7)
Sexual orientation GBMSM	983 (97)‡	128 (93)§

*Patients were median 36 years of age for both years (IQR 30–44 y in 2022 and 29–43 y in 2023). IQR, interquartile range; GBMSM, gay, bisexual, and other men who have sex with men. †May 6–Sep 16, 2022 (*1*). ‡Obtained via enhanced surveillance questionnaires, which were complete for 31% of case-patients by September 18, 2022. §107 case-patients self-identified identifying as GBMSM plus 21 who were adult men without recorded information on sexual orientation and no travel to mpox-endemic countries in Central or West Africa but were presumed to be part of the outbreak.

Most case-patients identified as GBMSM (107/137 [78%]); another 21 were adult men without recorded information about sexual orientation and no travel to the mpox-endemic countries in Central or West Africa. We included all of them as part of the 2022–2023 global clade IIb mpox outbreak, totaling 128 case-patients. Of the 9 case-patients excluded from the subsequent analysis, 8 were directly or indirectly linked to an mpox-endemic country; sporadic cases were distributed across time, and case-patients were heterosexual persons or children.

Most case-patients associated with the outbreak were HIV negative (69/128 [54%]), of whom most (51/69 [74%]) were taking HIV preexposure prophylaxis. Of the 128 HIV-negative case-patients, 28 (22%) reported attending an event involving sexual contact with multiple partners, and 24 (19%) had received a concurrent diagnosis of a sexually transmitted infection (Table 2).

**Table 2:** Characteristics and risk factors for case-patients with mpox confirmed or highly probable according to specimens collected in 2023, England*

Variable	No. (%), n = 128†
HIV status	
Living with HIV	13 (10)
HIV negative	69 (54)
Taking HIV PrEP‡	51 (74)
Not taking PrEP‡	18 (26)
Unknown	46 (36)
Modified Vaccinia Ankara–Bavarian Nordic vaccination status§	
Vaccinated: >2 doses¶	30 (23)
Vaccinated: 1 dose	20 (16)
Vaccinated: no. doses unknown	2 (2)
Unvaccinated	54 (42)
Unknown	22 (17)
Childhood smallpox vaccination status	
Vaccinated	4 (3)
Unknown	124 (97)
Hospital admission	
Yes	11 (9)
No	117 (91)
Unknown	
Reporting attending events involving multiple sex partners	
Yes	28 (22)
No	17 (13)
Unknown	83 (65)
Concurrent STI	
Yes	24 (19)
No	28 (22)
Unknown	76 (59)

*Patients were median 36 years of age, range 19–70 years, interquartile range 29–24 years. PrEP, preexposure prophylaxis; STI, sexually transmitted infection. †Unless otherwise stated, denominators are 128; denominators for percentage calculations are HIV-negative persons. ‡We do not have information on HIV treatment or outcomes of treatment for these cases. Nevertheless, most people living with HIV in England take antiretroviral therapy and have an undetectable viral load (*8*). §Estimated date of vaccination was available for 33 out of 52 vaccinated cases, all of whom received the Modified Vaccinia Ankara–Bavarian Nordic vaccine >2 weeks before infection. ¶Includes 1 person who reported receiving 3 doses.

Almost half of the case-patients with known vaccination status were fully or partially vaccinated (>1 dose) (52/106 [49%]), among whom 30 received >2 doses, 20 received 1 dose, and 2 did not report the number of doses. Among vaccinated case-patients, 20 (38%) reported attending an event involving sexual contact with multiple partners.

During July 2022–December 2023, a total of 77,543 GBMSM in England were vaccinated against mpox, 32,983 with 1 dose and 44,560 with 2 doses. MVA-BN vaccine coverage at the end of December 2023 was estimated at 37% for 1 dose and 50% for 2 doses (87% for 1 or 2 doses). Most vaccines had been given by March 2023 (91% first doses and 76% second doses).

VE of 1 dose was estimated at 84% (95% CI 74%–91%) (Table 3), comparable to the previous estimate of 78% (*7*). The VE of 2 doses was marginally, but not statistically significantly, lower than 1 dose, at 80% (95% CI 69%–83%) (p = 0.40). Overall VE of 1 or 2 doses was 82% (95% CI 74%–88%). Sensitivity analysis resulted in VE estimates that were markedly lower (Table 3), demonstrating that the method is affected by the estimated GBMSM population size.

**Table 3:** Vaccination status of mpox case-patients used to estimate vaccine effectiveness of various doses of Modified Vaccinia Ankara–Bavarian Nordic vaccine by using the screening method, England, 2023*

Doses	Cases in 2023	Corrected cases†	PCV, %		PPV, %	PPV, sensitivity, %‡	VE, % (95% CI)	VE sensitivity, % (95% CI)‡
0	54	65.4	52		16	30		
1	20	24.2	19		38	32	84 (74–91)	65 (42–79)
2	30	36.4	29		45	38	80 (69–83)	56 (31–72)
Unknown	22							
1 or 2	50	60.6	48		84	70	82 (74–88)	60 (41–73)
Total	126	126						

*PCV, percentage of case-patients with the given number of doses; PPV, percentage of the population with the given number of doses obtained by matching each case to the population uptake 14 d before onset and averaging this across cases; VE, vaccine effectiveness. †22 case-patients with unknown vaccination status distributed among 0, 1, and 2 doses. ‡Based on the higher estimated denominator for gay, bisexual, men who have sex with men (107,088).

Among known vaccinated case-patients in 2023, none were hospitalized. Of 11 (9%) persons who required hospital treatment for mpox, 9 were unvaccinated and vaccination status was unknown for 2.

## Conclusions

The low numbers of mpox cases in 2023 were initially interpreted as the final few cases of the 2022 outbreak (*1*). However, throughout the year, cases continued steadily, split evenly between imported infections and community transmission. The demographic and behavioral characteristics of mpox case-patients in 2023 remained comparable to those in 2022 (Table 1), indicating that mpox continues to circulate predominately within GBMSM sexual networks.

Nearly half of outbreak case-patients in 2023 were vaccinated, and there were more cases among those who had received 2 doses of MVA-BN vaccine than among those who had received 1 dose. Our analysis, based on full-year data from 2023, demonstrates that VE of 1 or 2 doses remains high (82%). The estimated VE for 2 doses compared with 1 dose was marginally lower, but the difference was not statistically significant. Considering that first doses will have been given farther in the past than second doses and that 2 doses would be expected to confer more protection, that finding is counterintuitive and may reflect differences in risk behavior among those who came forward for a second dose because they may also be at greater risk for exposure to monkeypox virus. Our observation is consistent with reports from other high-income countries with outbreaks predominantly among GBMSM. In May 2023, the Chicago Department of Public Health (Chicago, IL, USA) noted that most of the cases reported since mid-April were among men who had received 2 doses of MVA-BN vaccine (*9*), yet a higher number of first doses had been given compared with second doses overall (*10*).

We found that no vaccinated persons had been hospitalized for mpox in 2023, indicating that the MVA-BN vaccine probably protects against severe disease requiring hospitalization. Our finding is corroborated by a global case series that found illness among vaccinated persons to be less severe (*11*).

Among the limitations of our analysis, we were unable to examine VE in different population groups, because of unavailability of corresponding disaggregated coverage data. In addition, hospitalization resulting from clinical need was used as a proxy for severity, a pragmatic decision based on the unavailability of data using an objective measure.

Overall, the experience in England during 2023 was of continued low-level community transmission among GBMSM, as well as imported infections, which will probably continue. Given that ≈20 countries continued to report mpox cases in December 2023 and the World Health Organization assessment that the overall global risk for GBMSM remains moderate (*12*), continued low-level transmission is likely before elimination is reached. Our findings highlight the value of continued active prevention through vaccination and health promotion to persons at higher risk and ongoing surveillance to examine factors that contribute to continued transmission.

Appendix Additional results from study of the epidemiology of mpox and vaccine effectiveness, England, 2023.
