# Supplementary material for: Mpox Epidemiology and Vaccine Effectiveness, England, 2023
Source: Emerg Infect Dis. 2024 Oct;30(10):2145–8. doi: 10.3201/eid3010.240292 (PMC11431895; doi:10.3201/eid3010.240292)
Supplement: Appendix — Additional results from study of the epidemiology of mpox and vaccine effectiveness, England, 2023. [file 24-0292-Techapp-s1.pdf]

Article DOI: https://doi.org/10.3201/eid3010.240292

EID cannot ensure accessibility for supplementary materials supplied by authors. Readers who have difficulty accessing supplementary content should contact the authors for assistance.

Epidemiology of Mpox and Vaccine Effectiveness, England, 2023

Appendix

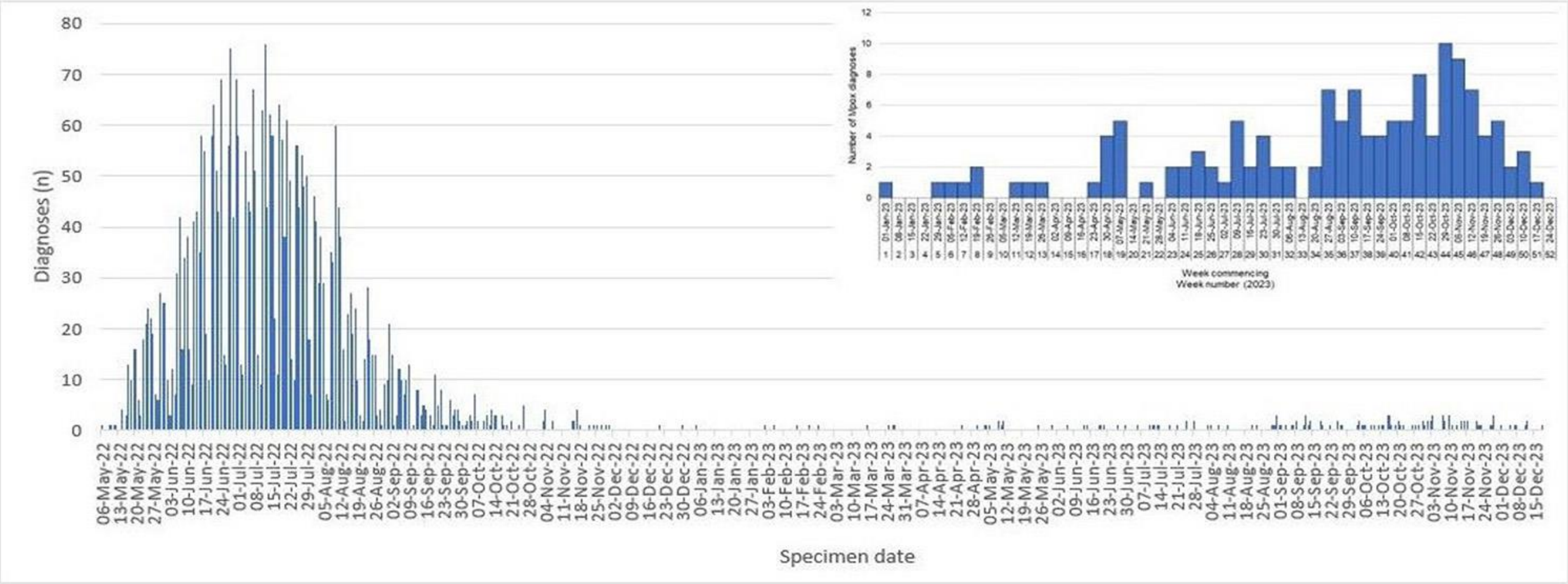

Appendix Figure. Confirmed and highly probable mpox cases by week of specimen date in England, May 6, 2022–December 31, 2023. Inset: Data by week of specimen date for 2023.
